# Case report: Implantation metastasis following stereotactic biopsy of pineal parenchymal tumor of intermediate differentiation in an adult patient: An exceptionally rare complication

**DOI:** 10.3389/fneur.2022.1019955

**Published:** 2022-11-17

**Authors:** Steven Tandean, Andre Marolop Pangihutan Siahaan, Michael Lumintang Loe, Rr Suzy Indharty, Julijamnasi, Mega Sari Sitorus, Iskandar Japardi, Julius July

**Affiliations:** ^1^Department of Neurosurgery, Faculty of Medicine, Universitas Sumatera Utara, Medan, Indonesia; ^2^Medical Faculty of Universitas Palangka Raya/Siloam Hospital Palangka Raya, Palangkaraya, Indonesia; ^3^Faculty of Medicine, Universitas Methodist Indonesia, Medan, Indonesia; ^4^Faculty of Medicine, Universitas Sumatera Utara, Medan, Indonesia; ^5^Department of Neurosurgery, Faculty of Medicine, Universitas Pelita Harapan, Tangerang, Indonesia

**Keywords:** stereotactic biopsy, pineal parenchymal tumor of intermediate differentiation, complication, metastase, implantation

## Abstract

Implantation metastasis following stereotactic biopsy in the brain had been reported as a rare complication. A 36-years-old female patient was treated with ventriculoperitoneal (VP) shunt and stereotactic biopsy of a pineal parenchymal tumor of intermediate differentiation (PPTID) with hydrocephalus. The patient underwent five cycles of radiotherapy on the pineal area. Seven years after the procedure, the patient developed left hemiparesis with the brain MRI findings showing an enhanced mass along the biopsy tract. Craniotomy tumor removal was carried out and the pathological assessment was consistent with those of the PPTID. Radiation on metastase area and craniospinal was subsequently performed. The patient was disease-free during the 2-year follow-up assessments. The potential occurrence of implantation metastasis following the stereotactic biopsy of PPTID should be considered in the treatment plan and follow-up assessments and evaluations. Expanding the radiation area to cover the entire biopsy tract may be favorable to lower the risk of implantation metastasis.

## Introduction

Pineal parenchymal tumors (PPTs) are rare and comprising of less than 0.3% of intracranial tumors. PPTs have heterogenous morphological and histological characteristics. Pineal parenchymal tumor of intermediate differentiation (PPTID) was first described by Schild et al. (1) and recently categorized as PPT WHO grade II or III according to 2007 WHO classification for tumors of the central nervous system (CNS). Several studies reported that PPTID is approximately 10–20% of the tumors of the pineal region (1–3).

Stereotactic biopsy for pineal region tumors should be selective, although is often considered relatively safe. This procedure has a high success rate of between 70 and 98% with a relatively low complication rate of between 0.5 and 4%. The complications include infection, hemorrhage, and neurological deficits (4, 5). Another rare complication after stereotactic biopsy is tumor seeding on the biopsy tract, as reported for several cases like pineal germinoma, craniopharyngioma, pineoblastoma, glioblastoma multiforme, brain metastase, and anaplastic astrocytoma (6–9).

To the best of our knowledge, the present case is the first case of metastatic seeding of the stereotactic biopsy tract trajectory of PPTID which occurred within 7 years following the procedure.

## Case report

**Figure 1:**
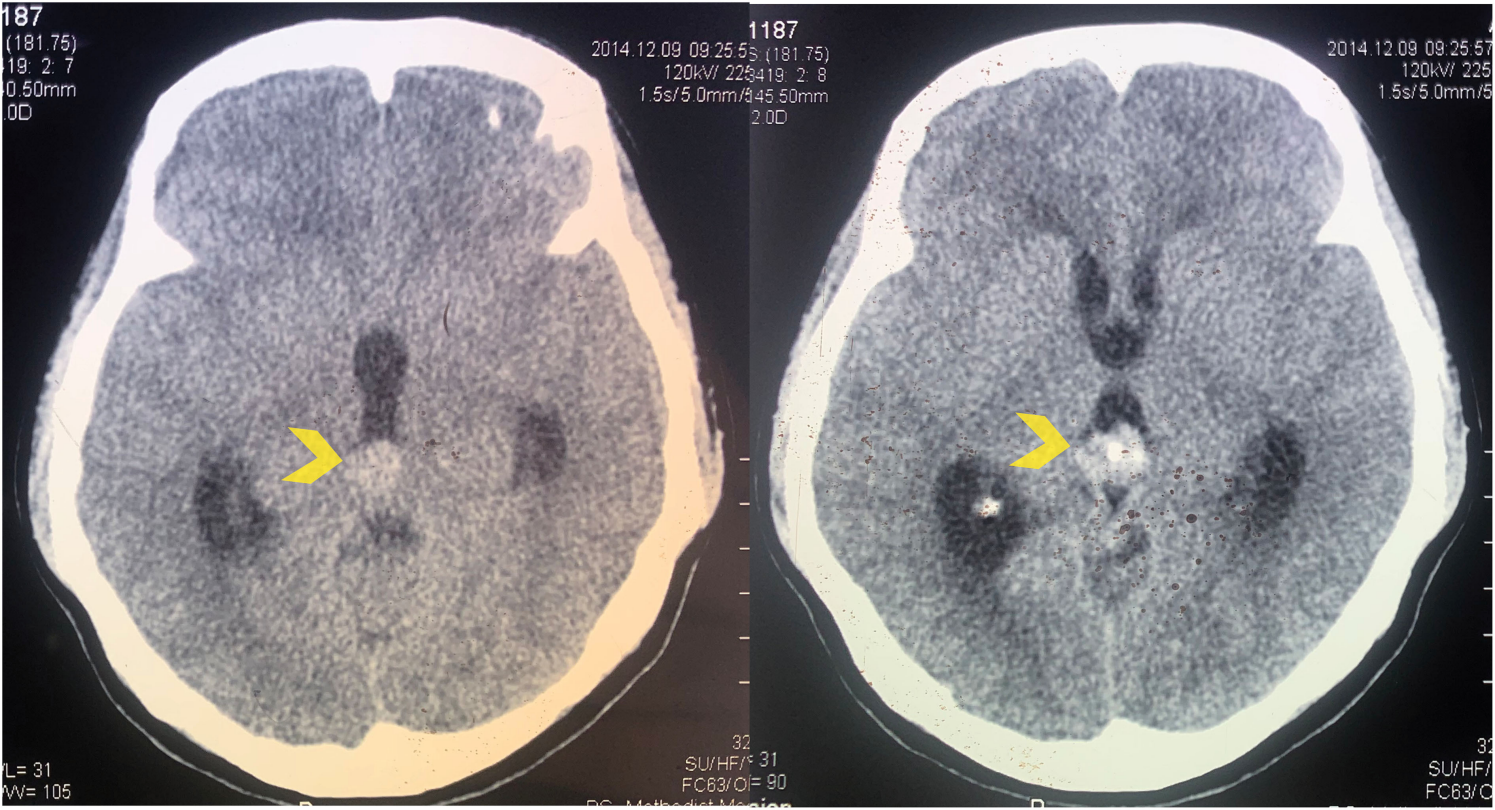
Axial CT scan without contrast showing the pineal region tumor at the time of the initial diagnosis in 2014 (yellow arrowhead).

A 36-year-old female patient presented with weakness and numbness in the left side limb. She recalled that the symptoms started 3 months prior and worsened gradually. Physical examination showed motoric strength of one-fifth on the left side. The patient had a history of pineal region tumor and hydrocephalus which were diagnosed 7 years prior from another hospital (Figure 1). Patient was initially treated with ventriculoperitoneal (VP) shunt to treat acute hydrocephalus then stereotactic biopsy with diagnosis result was a pineal parenchymal tumor of intermediate differentiation (PPTID). Cerebrospinal fluid (CSF) analysis revealed no tumor seeding. The patient then only underwent radiotherapy on the pineal region with a total of 45 Gy divided into 25 cycles.

**Figure 2:**
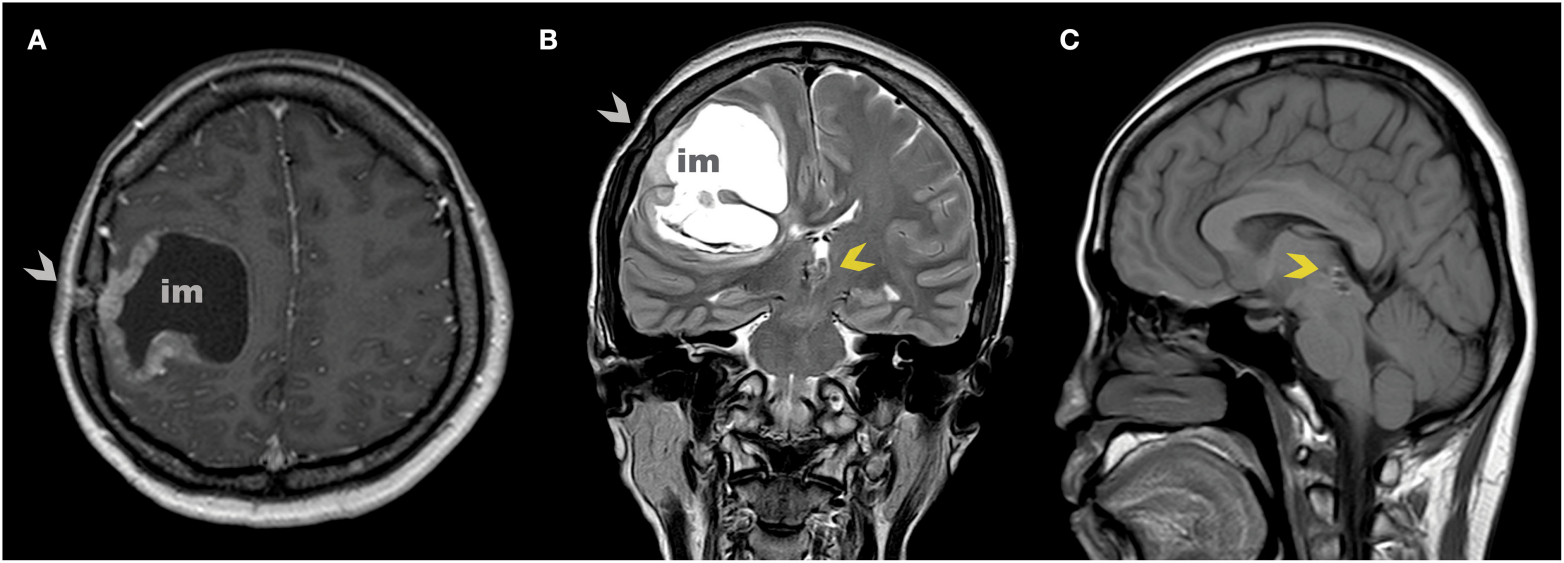
Axial **(A)** and coronal **(B)** MR scan at 7 years after the initial diagnosis show implantation metastasis under burr hole post stereotactic biopsy (gray arrowhead). Sagittal **(C)** MR scan shows no tumor on the pineal region (yellow arrowhead).

**Figure 3:**
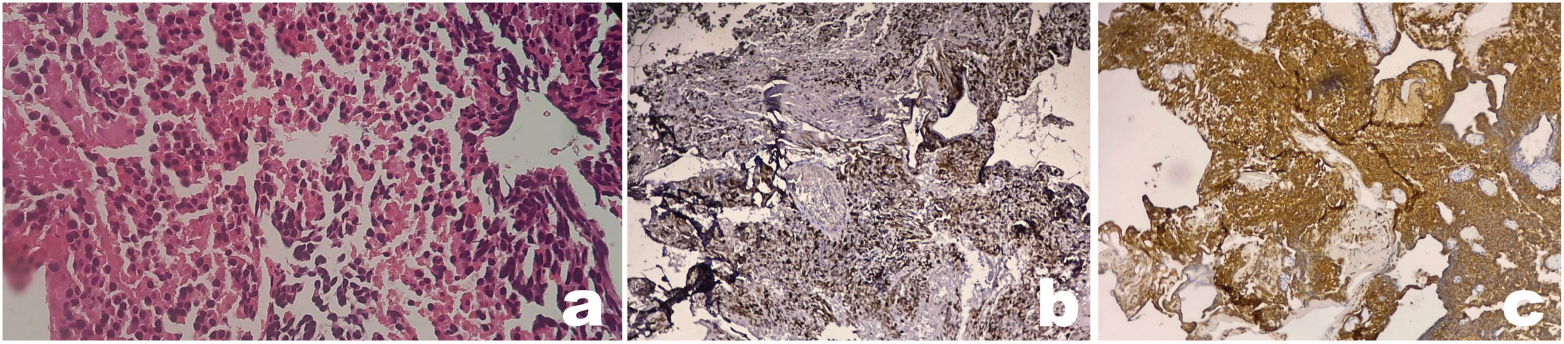
Pathology from implantation metastasis shows a typical pineal parenchymal tumor of intermediate differentiation (PPTID). Microscopy examination of tumor showed round-shaped cells with vesicular hyperchromatin, scanty eosinophilic cytoplasm, and nuclear atypia **(a)**. Immunohistochemical analyses show positive expression of synaptophysin **(b)** and chromogranin **(c)** (x100 magnification).

A brain MRI with IV contrast was then performed showing an isointense lesion with cystic parts that were slightly enhanced with contrast administration right below the burr hole site located in the right frontoparietal region. The lesion appeared to be causing a mass effect with the shifting of the midline (Figure 2). Craniotomy with near-total tumor removal was achieved without any eventful postoperative care. Histopathological examination showed round-shaped cells with vesicular hyperchromatin, scanty eosinophilic cytoplasm, nuclear atypia, and mitotic count of >4/10 hpf. Additionally, immunohistochemical analyses of Ki-67 showed synaptophysin and chromogranin expression. The Ki-67 index was 25%. The resulting final pathological assessment was PPTID (Figure 3).

**Figure 4:**
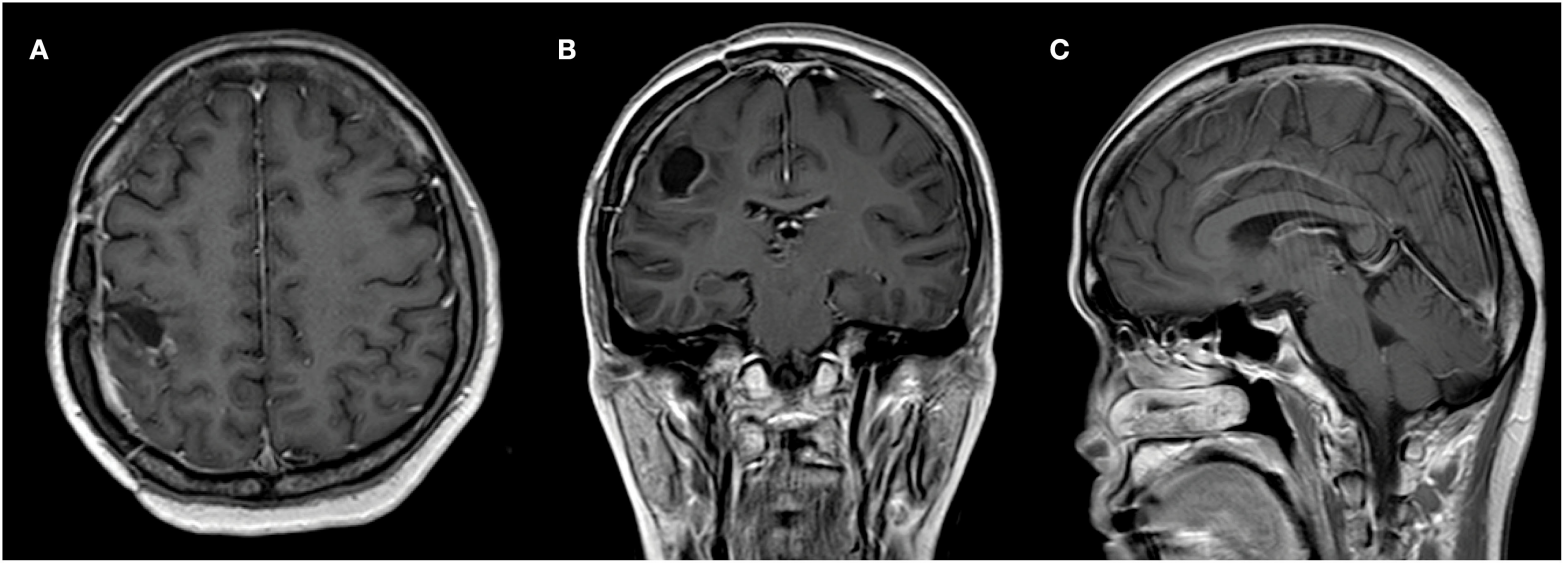
Axial **(A)**, coronal **(B)**, and sagittal **(C)** MR scan with contrast showing no tumor growth at the 2-year follow-up.

After the implantation, metastatic tumor removal surgery was performed. The motoric strength of the patient improved gradually and the patient was discharged on day 7. The external beam radiation with a 1.8 Gy fraction dose for 30 cycles to the tumor bed with volumetric modulated arc therapy (VMAT) and the craniospinal radiation was done 1 month after the surgery without the combination chemotherapy due to the patient's refusal. The follow-up brain and spine MRI scans were subsequently performed at 6 and 12 months. Brain MRI results showed gliosis at the surgical site without any sign of the residual tumor. The follow-up MRI was planned to be carried out every year thereafter. The patient was still disease-free at the latest follow-up at the 2-year mark (Figure 4).

## Discussion

In the present case, the patient was diagnosed with an implantation metastatic tumor of PPTID, 7 years after undergoing the stereotactic tumor biopsy for the initial diagnosis of PPTID. On presentation, the patient had a primary symptom of left-sided paralysis. The resulting brain imaging showed a cystic lesion on the right frontoparietal region just inferior to the prior burr hole. Surgery was then performed to remove the lesion and the following histological examination confirmed the similar features of PPTID.

The interesting radiographic feature that was observed in the patient was cystic-like lesions. These cystic-like lesions are features that are rarely seen in the PPTID (and its resulting metastases) radiographs (10, 11). Chatterjee et al. (12) proposed pathological prognostic factors in PPTID where tumors with a mitotic score of < 4/10 hpf and Ki-67 <5% are categorized as low grade, whereas tumors with a mitotic score of > 4/10 hpf and Ki-67 >5% are categorized as high grade (12). Therefore, we categorized the patient, with a mitotic score of >4/10 hpf and Ki-67 of about 25%, as a high grade with a relatively high probability of recurrences. Consequently, the follow-up MRI was planned to be carried out every year.

The gross total resection followed by radiotherapy and chemotherapy is the ideal treatment for PPTID (13). Due to the location of the pineal body, a stereotactic needle biopsy is an effective option in establishing the diagnosis of a pineal region tumor. The needle track seeding metastasis after the tumor biopsy is rare and probably associated with incidental implantation, which most likely occurs during the withdrawal of the biopsy needle. There had been documented cases of seeding metastasis after the biopsies of the lung, kidney, prostate, liver, pancreas, and thyroid carcinoma. Moreover, the tumors of the central nervous system have a risk of metastasis on its biopsy tracks which have been documented in pineoblastoma, glioblastoma, anaplastic astrocytoma, and brain metastases. Therefore, histological assessments and subsequent diagnoses of the brain lesions are mandatory for establishing appropriate treatment plans (5, 14).

The secondary lesions that occurred, in this case, developed right below the burr hole site along the stereotactic trajectory tract with no evidence of residual tumor at the pineal region on MR imaging. We suspect this was the cause of the implantation metastasis. To our knowledge, this is the first report of PPTID with implantation metastases along the trajectory tract following the biopsy procedure of the pineal region. There were only two previously reported cases of implantation metastases along the trajectory tract but were associated with pineal germinoma and pineoblastoma (6, 14). Haw and Steinbok (6) reported an implantation metastasis along the ventriculostomy biopsy tract outside the radiotherapy area in a 14-year-old male patient suffering from a pineal germinoma. The gross total removal of the metastatic mass was performed by craniotomy which was then followed by a chemotherapy course. A 12-month follow-up showed no recurrence (6). Furthermore, Rosenfeld et al. (14) reported a pineoblastoma in an 18-month-old which was confirmed with the transfrontal stereotactic biopsy and subsequently treated with chemotherapy. An implantation metastatic tumor along the biopsy tract was found 13 weeks later which was then treated with a more aggressive regimen. Unfortunately, the patient died at week 32 due to the partial response with immediate recurrence after the chemotherapy was stopped (14).

The pineal parenchymal tumor of intermediate differentiation is a rare central nervous system tumor (<1%) that arises from the pineal parenchyma. The spectrum is between pineocytoma (grade I) and pineoblastoma (grade IV). Due to its rarity and heterogenous behavior, there is still no consensus regarding the optimum treatment. The systematic review by Mallick et al. (15) reported that adjuvant radiotherapy in PPTIDs was favorable and could result in higher survival outcomes (15). Additionally, expanding the radiation area to include biopsy tracts may be beneficial to avoid the occurrence of implantation metastases, illustrated by the present case. Furthermore, Boutin et al. (16) found that, in malignant pleura mesothelioma cases, none of the patients that were subjected to the radiation on the biopsy tract following the needle biopsy using thoracoscopy developed implantation metastases. Conversely, 40% of the patients that did not receive biopsy tract radiation following needle biopsy using thoracoscopy developed implantation metastases (16).

## Conclusion

Although it is rare, the potential occurrence of implantation metastasis following the stereotactic biopsy of PPTID should be considered in the treatment plans and follow-up. Considering the area and doses of radiation on PPTID, particularly on the biopsy track, may be advantageous, but additional research is required.

## Data availability statement

The original contributions presented in the study are included in the article/supplementary material, further inquiries can be directed to the corresponding author.

## Ethics statement

Written informed consent was obtained from the individual(s) for the publication of any potentially identifiable images or data included in this article. The board of the Siloam Dhirga Surya Hospital's Ethics Committee (Medan, Indonesia) evaluated and approved this work (No. 0625/SHMD/DIR/SB/V/2022).

## Author contributions

ST and AS: conception, design, and reviewed submitted version of manuscript. ST, Julijamnasi, MS, AS, and RI: acquisition of data. ST, Julijamnasi, MS, and AS: analysis and interpretation of data. ST and ML: drafting the article. JJ and IJ: critically revising the article. ST: approved the final version of the manuscript on behalf of all authors.

## Conflict of interest

The authors declare that the research was conducted in the absence of any commercial or financial relationships that could be construed as a potential conflict of interest.

## Publisher's note

All claims expressed in this article are solely those of the authors and do not necessarily represent those of their affiliated organizations, or those of the publisher, the editors and the reviewers. Any product that may be evaluated in this article, or claim that may be made by its manufacturer, is not guaranteed or endorsed by the publisher.
